# A race against time – an evaluation of lifesaving treatment pathways for patients following cardiac arrest in a non-hospital setting.

**DOI:** 10.23889/ijpds.v9i5.2599

**Published:** 2024-09-18

**Authors:** Rowena Bailey, Laura Herbert, Richard Pugh, Ronan Lyons, Tamas Tszakmany

**Affiliations:** 1 Swansea University; 2 Department of Anaesthetics, Glan Clwyd Hospital, NHS Wales; 3 Department of Anaesthesia, Intensive Care and Pain Medicine, Division of Population Medicine, Cardiff University

## Background

Cardiac arrest occurs when the heart ceases pumping blood around the body. Most take place outside hospital. A sequence of effective interventions (a “chain of survival”) is essential for patient survival and recovery of organ function. Out of Hospital Cardiac Arrest (OHCA) requires prompt recognition and call for help, proficient CPR, early defibrillation and conveyance by the emergency services to an appropriate hospital. If initial resuscitation is successful, effective post-resuscitation care is required, delivered usually in the intensive care unit (ICU). New patient pathways were introduced following an update to national guidelines, which recommend conveyance of patients to centres with percutaneous coronary intervention (PCI) services where possible.

## Objective

We have evaluated outcomes for patients, comparing those conveyed to PCI settings and those conveyed elsewhere to quantify the effect of the new patient pathway on mortality, survival and length of hospital stay.

## Approach

We have linked electronic health records from emergency departments, acute hospital settings, ICUs and the national deaths register for OHCA patients living in Wales, UK. We have undertaken temporal analysis and time to event analysis to compare patient subgroups.

## Results

Variation in patient pathways will be described using characteristics of conveyance method, the type of treating hospital and transfers between PCI and non-PCI settings adjusted for individual patient level confounders such as age, sex and comorbidities.

## Conclusion

This work is important to evaluate efficacy of operational procedures and inform national policies which aim to improve survival following OHCA and save lives.

